# A loss-of-function mutation in *KCNJ11* causing sulfonylurea-sensitive diabetes in early adult life

**DOI:** 10.1007/s00125-024-06103-w

**Published:** 2024-02-17

**Authors:** Natascia Vedovato, Maria V. Salguero, Siri Atma W. Greeley, Christine H. Yu, Louis H. Philipson, Frances M. Ashcroft

**Affiliations:** 1 Department of Physiology, Anatomy and Genetics, University of Oxford, Parks Road, Oxford, UK; 2 Departments of Medicine and Pediatrics, Section of Endocrinology Diabetes and Metabolism, University of Chicago, Chicago, IL USA; 3 Division of Endocrinology, Department of Pediatric Medicine, St Jude Children’s Research Hospital, Memphis, TN USA

**Keywords:** Congenital hyperinsulinism, Glibenclamide, K_ATP_ channel, Pharmacological chaperone

## Abstract

**Aims/hypothesis:**

The ATP-sensitive potassium (K_ATP_) channel couples beta cell electrical activity to glucose-stimulated insulin secretion. Loss-of-function mutations in either the pore-forming (inwardly rectifying potassium channel 6.2 [Kir6.2], encoded by *KCNJ11*) or regulatory (sulfonylurea receptor 1, encoded by *ABCC8*) subunits result in congenital hyperinsulinism, whereas gain-of-function mutations cause neonatal diabetes. Here, we report a novel loss-of-function mutation (Ser118Leu) in the pore helix of Kir6.2 paradoxically associated with sulfonylurea-sensitive diabetes that presents in early adult life.

**Methods:**

A 31-year-old woman was diagnosed with mild hyperglycaemia during an employee screen. After three pregnancies, during which she was diagnosed with gestational diabetes, the patient continued to show elevated blood glucose and was treated with glibenclamide (known as glyburide in the USA and Canada) and metformin. Genetic testing identified a heterozygous mutation (S118L) in the *KCNJ11* gene. Neither parent was known to have diabetes. We investigated the functional properties and membrane trafficking of mutant and wild-type K_ATP_ channels in *Xenopus* oocytes and in HEK-293T cells, using patch-clamp, two-electrode voltage-clamp and surface expression assays.

**Results:**

Functional analysis showed no changes in the ATP sensitivity or metabolic regulation of the mutant channel. However, the Kir6.2-S118L mutation impaired surface expression of the K_ATP_ channel by 40%, categorising this as a loss-of-function mutation.

**Conclusions/interpretation:**

Our data support the increasing evidence that individuals with mild loss-of-function K_ATP_ channel mutations may develop insulin deficiency in early adulthood and even frank diabetes in middle age. In this case, the patient may have had hyperinsulinism that escaped detection in early life. Our results support the importance of functional analysis of K_ATP_ channel mutations in cases of atypical diabetes.

**Graphical Abstract:**

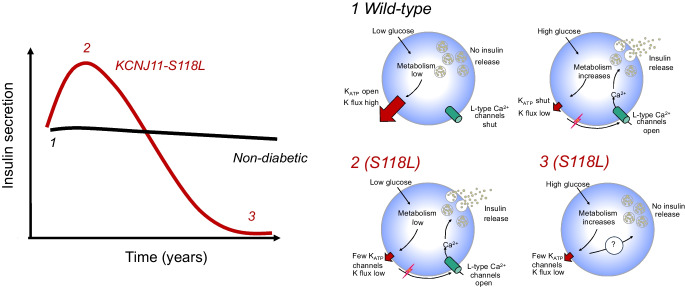


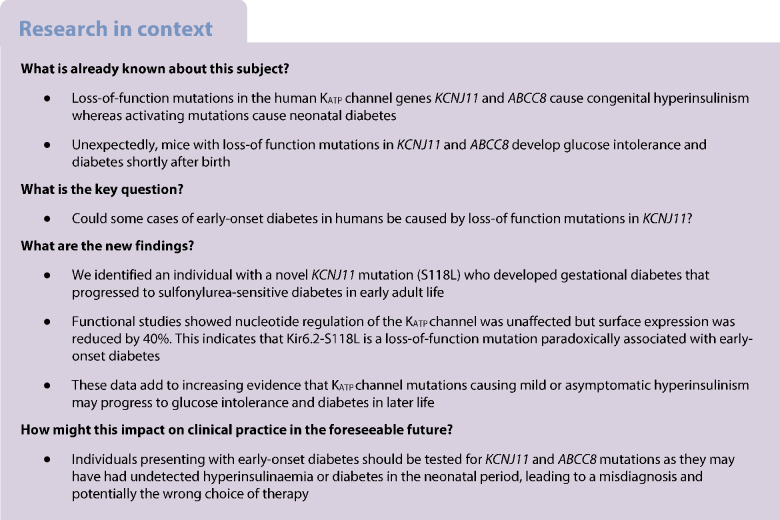


## Introduction

The central role of the ATP-sensitive potassium (K_ATP_) channel in glucose-stimulated insulin secretion from pancreatic beta cells is well understood [1]. Glucose uptake and metabolism leads to an increase in the ATP/ADP concentration that causes a reduction in K_ATP_ channel activity. This triggers electrical activity and elevation of the cytosolic calcium concentration that initiate insulin granule exocytosis, thereby linking glucose metabolism to electrical activity. Mutations in either the pore-forming subunit (the inwardly rectifying potassium channel 6.2 [Kir6.2], encoded by *KCNJ11*) or the regulatory subunit (the sulfonylurea receptor 1 [SUR1], encoded by *ABCC8*) of the K_ATP_ channel hetero-octameric complex cause human disease. Loss-of-function mutations cause persistent and unregulated insulin secretion and congenital hyperinsulinism [2–4], whereas gain-of-function mutations prevent K_ATP_ channel closure in response to glucose metabolism, resulting in a failure of insulin secretion and neonatal diabetes [5, 6]. Sulfonylurea drugs, widely used to treat type 2 diabetes, bypass metabolism and close the K_ATP_ channel directly, thus stimulating insulin release in both neonatal and type 2 diabetes [7].

Recessively inherited loss-of-function mutations in either *ABCC8* or *KCNJ11* are the most common cause of congenital hyperinsulinism [8, 9]. They principally result in a failure of K_ATP_ channel assembly or trafficking to the cell surface. Diazoxide therapy is often ineffective because of the absence of K_ATP_ channels, which may necessitate partial pancreas resection to control the hypoglycaemia. This inevitably leads to diabetes in later life and has prompted a more conservative approach to disease management in recent years. Several small molecules have been shown to correct channel biogenesis and trafficking defects but none are yet used clinically [10–12]. K_ATP_ mutations causing dominantly inherited hyperinsulinism are less common than recessive mutations. They generally produce channels that traffic normally to the cell surface but have altered gating or impaired opening in response to MgADP [2, 3]. This results in a milder hypoglycaemia phenotype that is usually responsive to diazoxide or can be managed by diet, and may even escape detection. Adult carriers are frequently asymptomatic unless challenged with fasting or protein tolerance tests [13, 14].

It is becoming increasingly evident that individuals who have hyperinsulinism caused by dominant K_ATP_ channel mutations may show lessening of symptoms with age and progress to spontaneous remission, glucose intolerance or even diabetes [13–23]. It has been argued that the incidence of diabetes in individuals with hyperinsulinism is no greater than that in the general population [13]. However, this has been contested [14]. It is noteworthy that in one Finnish family, of the 11 family members who were heterozygous carriers of a loss-of-function K_ATP_ channel mutation (*ABCC8*-E1506K), all reported symptoms of hypoglycaemia in childhood, four had overt diabetes and five had glucose intolerance with severely blunted first-phase glucose-stimulated insulin secretion in adult life [15], an incidence substantially higher than in the general population. In contrast, the frequency of diabetes in a second family with this mutation was comparable to that of the general population [13]. However, subsequent studies have provided additional support for an association between hyperinsulinism in childhood and glucose intolerance, gestational diabetes and diabetes in later life [14, 16–23]. Clearly, this possibility requires further investigation, both because it may shed light on the aetiology of diabetes and because of its implications for therapy.

In this paper, we therefore study the functional effects of a dominant loss-of function *KCNJ11* mutation that did not cause detectable hyperinsulinism in infancy but was associated with sulfonylurea-sensitive diabetes in the absence of an autoimmune or other monogenic cause, in early adult life.

## Methods

### Ethics statement

Our research studies followed the University of Chicago Institutional Review Board protocols (http://monogenicdiabetes.uchicago.edu). Written informed consent was obtained from the patient for publication of the submitted article.

### Molecular genetics

Next-generation sequencing (NGS) panel for monogenic diabetes, including 14 monogenic diabetes-related genes (*ABCC8*, *APPL1*, *BLK*, *CEL*, *GCK*, *HNF1A*, *HNF1B*, *HNF4A*, *INS*, *KCNJ11*, *KLF11*, *NEUROD1*, *PAX4*, *PDX1*) and deletion/duplication analysis of the coding region of the same panel of genes, was performed by array-CGH at the University of Chicago Services laboratory.

### Chemicals and antibodies

Table 1 gives the source of chemicals and antibodies used. Antibodies have been extensively validated by the companies and in numerous publications.

**Table 1:** Source of reagents and antibodies

Reagent / antibody	Catalogue no.	Concentration and storage buffer	Source
Reagents			
Glibenclamide	G0639	NA	Sigma-Aldrich, UK
Tolbutamide	T091	NA	Sigma-Aldrich, UK
Diazoxide	D9035	NA	Sigma-Aldrich, UK
Na-azide	S-8032	NA	Sigma-Aldrich, UK
MgATP	A9187	NA	Sigma-Aldrich, UK
Antibodies			
Anti-HA (rat)	11867423001	0.2 μg/ml in ddH_2_O	Roche Products, UK
Goat anti-rat IgG	112-036-003	0.8 mg/ml in ddH_2_O	Jackson ImmunoResearch, UK
α-Tubulin	2144	29 μg/ml in PBS	Cell Signaling Technology, USA
Donkey anti-rabbit	NA934V	0.18 mg/ml in PBS	Avantor (VWR), UK

### Molecular biology and cell preparation

pcDNA4/TO or pBF expression vectors were used to express the human Kir6.2 or SUR1 genes (*KCNJ11* and *ABCC8*, respectively). The QuikChange XL system (Stratagene, UK) was used for site-directed mutagenesis, and mutant clones were verified by sequencing (SourceBioScience, UK). SP6 mMESSAGE MACHINE transcription kit (catalogue no. AM1340, ThermoFisher, UK) was used for mRNA synthesis [24].

HEK-293T cells were obtained from LGC Standards, UK (ATTC CRL-3216). Our working stock tested negative for mycoplasma contamination (MycoAlert Mycoplasma Detection Kit, Lonza Bioscience, UK). Cells were maintained at 50% to 90% confluency at 37°C, and 5% CO_2_/95% air, in DMEM (4.5 g/l glucose; Sigma, UK), supplemented with 10% (vol./vol.) FBS (Life Technologies, UK), 100U/ml penicillin and 100µg/ml streptomycin (ThermoFisher Scientific, UK). When close to confluency, cells were gently dissociated with TryPLE (ThermoFisher Scientific) and seeded in 25 cm^2^ flasks. After 3 h, cells were transfected with 4.5 µg of wild-type *SUR1* cDNA and 1.5 µg of either wild-type *KCNJ11* cDNA, mutant *KCNJ11* cDNA (homozygous [hom]), or a 50:50 mixture of wild-type and mutant *KCNJ11* cDNAs (to simulate the heterozygous [het] state of the patient), using TransIT-LT1 (Mirus Bio, USA). Where indicated, 5 µmol/l glibenclamide was added to the media just before transfection. After 48 h, transfected cells were plated onto 35 mm petri dishes coated with poly-l-lysine (Corning, UK) and currents were recorded 24 h later.

*Xenopus* oocytes were prepared as previously described [25], injected with 0.8 ng wild-type or mutant *KCNJ11* mRNA and ∼4 ng *SUR1* mRNA, and maintained in Barth’s solution (in mmol/l: 88 NaCl, 1 KCl, 1.68 MgSO_4_, 0.41 CaCl_2_, 0.47 Ca[NO_3_]_2_, 2.4 NaHCO_3_, 10 HEPES, adjusted to pH 7.4 with NaOH) at 18°C. Currents were recorded 2–4 days after injection.

### Electrophysiology

Giant inside-out patches were excised from HEK-293T cells expressing wild-type or mutant K_ATP_ channels. Macroscopic currents were recorded at −60 mV with an Axopatch 200B amplifier (Molecular Devices, UK), filtered at 1 kHz, and digitised at 10 kHz with a Digidata 1322A A/D interface driven by pClamp9 software (Molecular Devices). The extracellular (pipette) solution contained (in mmol/l) 140 KCl, 1.2 MgCl_2_, 2.6 CaCl_2_ and 10 HEPES, pH 7.4 with KOH. The intracellular (bath) solution contained (in mmol/l) 107 KCl, 2 MgCl_2_, 1 CaCl_2_, 10 EGTA and 10 HEPES (pH 7.3 with KOH), plus MgATP and/or MgADP as indicated.

For each MgATP concentration tested, current was expressed as a fraction of its mean value in the control intracellular solution before and after each ATP application [26]. The MgATP concentration producing half-maximal block of the K_ATP_ current (IC_50_) and the Hill coefficient were calculated from the Hill fit to each individual current–inhibition curve. For each construct, currents were recorded from at least four or five separate cells, and at least two independent transfections. However, the heterozygous channel recordings were from two cells. To test channel activation, 100 μmol/l MgADP was added in the presence of 100 μmol/l MgATP. Current was expressed as a fraction of the mean values in control solution (no nucleotides) before and after each application.

Whole-oocyte K_ATP_ currents were recorded at room temperature in response to ±20 mV steps from a holding potential of −10 mV using a two-electrode voltage-clamp (GeneClamp 500B amplifier; Molecular Devices). Data were filtered online at 500 Hz, digitised at 4 kHz with a Digidata 1440A acquisition system (Molecular Devices) and acquired with pCLAMP10 software (Molecular Devices). Oocytes were perfused with control solution containing (in mmol/l) 90 KCl, 1 MgCl_2_, 1.8 CaCl_2_ and 5 HEPES (adjusted to pH 7.4 with KOH). Na-azide (3 mmol/l), diazoxide (340µmol/l) or tolbutamide (500µmol/l) were added as indicated. Data were recorded from ‘*n*’ oocytes, from at least two separate frogs, and analysed with ClampFit (pClamp 11.2.0.59; Molecular Devices) and Prism 10.1.2 (GraphPad, USA).

### Surface expression

Surface expression of wild-type or mutant Kir6.2 was measured based on their ability to chaperone a site-specific tagged SUR1 to the plasma membrane [27]. Briefly, a haemagglutinin (HA) tag (YAYMEKGITDLAYPYDVPDYA) was inserted into the last extracellular loop of SUR1 (TM16-TM17). Transfections were performed as described above and 72 h post-transfection cells were rinsed with PBS, fixed for 30 min in 10% neutral buffered formalin, rinsed twice with PBS and blocked with PBS+1% BSA for 30 min at 4°C. Anti-HA monoclonal antibody was added for 1 h at 4°C at a 1:1000 dilution, followed by multiple washes on ice with PBS+1% BSA. Cells were then incubated with horseradish peroxidase-conjugated goat anti-rat polyclonal antibody (diluted 1:1000) for 30 min at 4°C. After multiple washes with PBS+1% BSA and PBS, 500 μl of SuperSignal ELISA Femto Maximum Sensitivity Substrate (ThermoFisher Scientific) was added to each 35 mm dish and the chemiluminescence signal was measured using a Glomax 20/20 Luminometer (Promega, UK) after a 10 s incubation. Total protein in each dish was determined by BCA (ThermoFisher Scientific) and used to normalise the chemiluminescent signal. Each experimental point is the mean of three dishes from one transfection. Data were collected from three to five independent transfections and plotted as a percentage of the expression of the wild-type channel, for each individual experiment.

### Western blotting

Western blotting was performed on total protein from HEK-293T cells transfected as described above (from three independent transfections), using an HA tag inserted into SUR1 to detect SUR1 abundance. Cells were harvested and solubilised in lysis buffer (0.5% Triton X-100, 100 mmol/l potassium acetate, pH 7.4), supplemented with cOmplete protease inhibitor cocktail (Roche Products, UK). Benzonase (Sigma) was added to each sample (250 U per 100 μl of solubilised cells) and incubated for 30 min at room temperature. Protein concentrations were measured by BCA and 20 µg of protein lysate was mixed with a reducing agent and loading buffer (NuPAGE; Invitrogen, UK). α-Tubulin was used as a control for equal loading. After running a precast NuPAGE 7% Tris-acetate poly-acrylamide gels at 150 V for 60 min, proteins were wet transferred overnight onto polyvinylidene difluoride membranes (Immobilon P; Merck Millipore, Germany) at 10 V in Tris-glycine buffer (25 mmol/l Tris, 192 mmol/l glycine, 10% methanol). Non-specific protein signal was blocked by incubating the membranes with 5% milk in TBS-T (150 mmol/l NaCl, 1% Tween 20, 25 mmol/l Tris, pH 7.2). Primary (1:1000 dilution) and secondary (1:20,000) antibodies were incubated for 30 min at room temperature, each time followed by multiple washes with TBS-T. Immunoreactive proteins were detected by chemiluminescence (SuperSignal West Pico Chemiluminescent Substrate; Thermo Fisher). Analysis was performed using ImageJ 1.53K (NIH, USA).

### Statistics

Results are reported as individual data points and mean±SEM. Statistical significance was determined using Student’s *t* test or one-way ANOVA followed by the post hoc Dunnett’s test for multiple comparisons.

## Results

### Clinical history

A previously healthy, non-obese (BMI 20–21 kg/m^2^) 31-year-old woman of white/European ancestry was diagnosed with mild hyperglycaemia and an HbA_1c_ of 38 and 39 mmol/mol (5.6 and 5.7%) during successive employee screens at her place of work. She was not aware of any symptoms of either hypoglycaemia or hyperglycaemia in early life. There was no history of diabetes in her parents or siblings but her father and two siblings had a history of obesity. Two years later, during her first pregnancy, she had persistently elevated blood sugar levels: 1 h OGTT of 12.8 mmol/l; HbA_1c_ of 42 mmol/mol (6.0%); and fasting blood glucose of 8.6 mmol/l at 27 weeks and 6 days of gestation. She was initially started on sulfonylurea therapy (glibenclamide 2.5 mg/day), which helped her glycaemic control, but her medication was subsequently switched to insulin.

The patient’s glycaemic control improved after her first pregnancy but did not recover completely, and she required insulin during her next two pregnancies (three pregnancies in total). Between her first and second pregnancies, and while off medication, her fasting insulin was 25 pmol/l and her C-peptide was 0.51 nmol/l (with fasting glucose of 6.7 mmol/l). Insulin was discontinued 6 months after the birth of her third child but she continued to experience increases in blood glucose (fasting glucose 7.3 mmol/l, HbA_1c_ 48 mmol/mol [6.5%]). She was therefore treated with glibenclamide (7.5 mg daily) plus metformin (1000 mg twice daily). At follow-up, her HbA_1c_ was 43 mmol/mol (6.1%), 88% of time within the target range, with a continuous glucose monitor report of modest hypoglycaemia (2% of the time) and hyperglycaemia (9% of the time). The following year, when aged 38 years, her HbA_1c_ was 56 mmol/mol (7.3%) while taking metformin (1000 mg at night) and glibenclamide (2.5–5 mg in the morning and 5 mg before the evening meal). Metformin was stopped due to gastrointestinal symptoms; oral semaglutide (Rybelsus, 3 mg daily) was started and glibenclamide was increased to 5 mg in the morning and 5 mg before dinner. At follow-up, the patient reported decreased appetite and mild weight loss (from 56 to 53 kg). Her HbA_1c_ was 48 mmol/mol (6.5%; 92% in range as measured by continuous glucose monitoring) without hypoglycaemia (<1%) and modest hyperglycaemia (7%), while taking glibenclamide (5 mg twice daily) and oral semaglutide (7 mg daily). All three of the patient’s children were delivered by C-section. Their gestational age and birth weights were as follows: 36 weeks 6 days, 3.43 kg; 38 weeks 1 day, 4.43 kg; and 38 weeks 2days, 3.98 kg.

### Molecular genetics

Given the young age of the patient (31 years at diagnosis), lack of islet cell antibodies and lack of obesity, a genetic origin for her insulin-independent diabetes was implicated. Genetic testing was undertaken at the age of 35 years using a panel of genes known to cause MODY. No mutations were found in *ABCC8*, *APPL1*, *BLK*, *CEL*, *GCK*, *HNF1A*, *HNF1B*, *HNF4A*, *INS*, *KCNJ11*, *KLF11*, *NEUROD1*, *PAX4* and *PDX1* but sequence analysis identified a novel heterozygous missense mutation, p.Ser118Leu (c.353C>T ), in exon 1 of the *KCNJ11* gene. This variant has been previously reported as a ‘variant of uncertain significance’ [4], although no functional studies were carried out. We were unable to obtain details of the clinical history of the individual studied in this earlier report but note that after starting sulfonylureas their HbA_1c_ dropped from 124 mmol/mol (13.5%) to 52 mmol/mol (6.9%). To evaluate whether the *KCNJ11* variant could explain our patient’s diabetes, her response to sulfonylureas and her lack of insulin resistance, we undertook functional analysis of the S118L mutation in *KCNJ11*. Unfortunately, none of her children have been tested for this variant but their blood glucose levels have remained within normal limits.

### The S118L mutation does not affect K_ATP_ channel ATP sensitivity

**Fig. 1:**
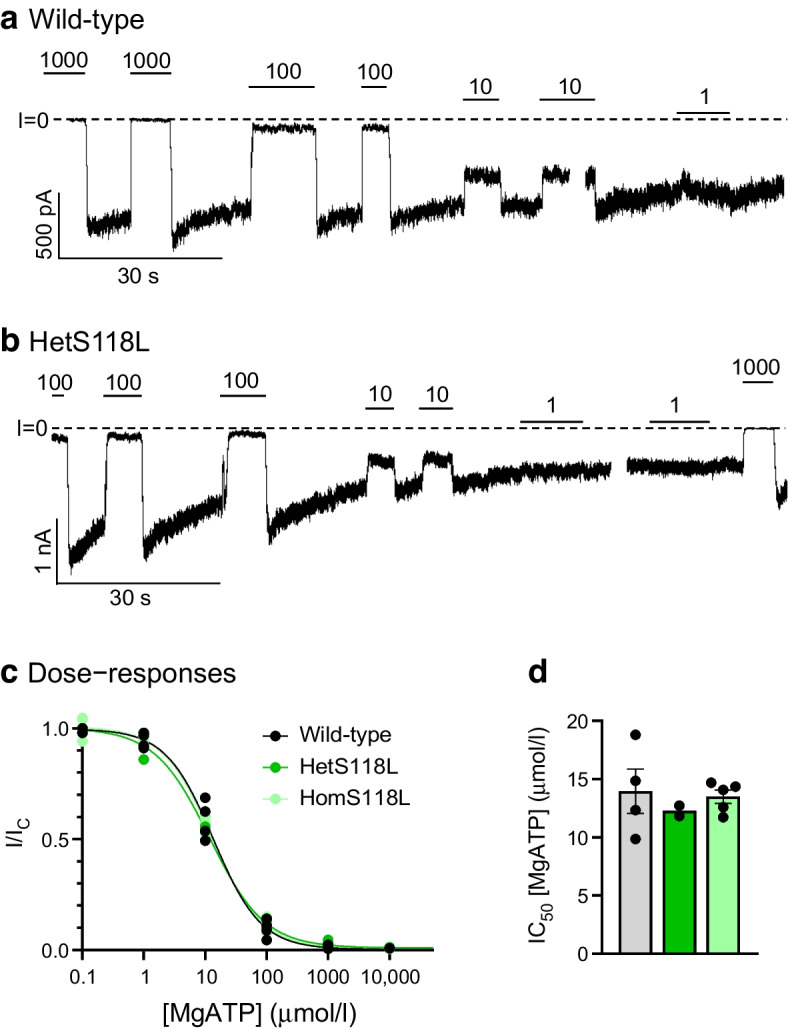
Inhibition of K_ATP_ currents by MgATP. (**a**, **b**) Representative current traces for wild-type (**a**) and hetS118L (**b**) channels recorded at −60 mV from inside-out patches excised from HEK293T cells and exposed to different MgATP concentrations (as indicated by the bars; values are in μmol/l). The zero-current level (I=0) is shown by the dashed lines. (**c**, **d**) MgATP concentration–response curves (**c**) and corresponding IC_50_ values (**d**) for wild-type (black/grey, *n*=4), hetKir6.2-S118L/SUR1 (hetS118L; dark green, *n*=2) and homKir6.2-S118L/SUR1 (homS118L; light green, *n*=5) channels. In (**c**), current amplitude (I) is expressed as a fraction of the maximum K_ATP_ current measured in control solution in the same patch (I_C_). The lines are the best fit of the Hill equation to the mean data. In (**d**), IC_50_ values were obtained from individual MgATP concentration–response curves. Box plots show individual data points and mean ± SEM

We examined whether the Kir6.2-S118L mutation affects the sensitivity of the K_ATP_ channel to MgATP inhibition by recording K_ATP_ currents in inside-out patches (Fig. 1a,b). HEK293T cells were co-transfected with wild-type SUR1 and either Kir6.2 (wild-type), Kir6.2-S118L (homS118L) or a 1:1 mixture of wild-type Kir6.2 and Kir6.2-S118L (hetS118L). The latter aims to simulate the heterozygous state of the patient. There was no significant difference in the dose–response curves for ATP inhibition or the corresponding half-maximal block (IC_50_) of wild-type (14±2 µmol/l, *n*=4), hetS118L (11.8–12.7 µmol/l, *n*=2) or homS118L (13.5±0.5 µmol/l, *n*=5) channels (Fig. 1c,d). However, the current immediately after patch excision was less for homS118L channels (738±279 pA, median 362 pA, *n*=8) than for wild-type channels (2639±911 pA, median 2238 pA, *n*=4).

### The S118L mutation does not affect K_ATP_ channel stimulation by MgADP

**Fig. 2:**
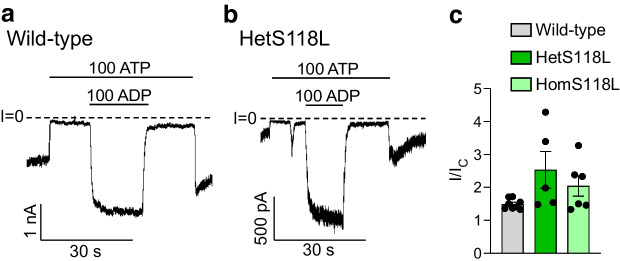
Activation of K_ATP_ currents by MgADP. (**a**, **b**) Representative current traces for wild-type (**a**) and hetKir6.2-S118L/SUR1 (**b**, hetS118L) channels recorded at −60 mV from inside-out patches excised from HEK293T cells and exposed to 100 μmol/l MgATP with or without 100 μmol/l MgADP (as indicated by the bars, values are in μmol/l). The zero-current level (I=0) is shown by the dashed lines. (**c**) Current amplitude (I) expressed as a fraction of the maximum K_ATP_ current measured in control solution in the same patch (I_C_) for wild-type (grey), hetS118L (dark green) and homS118L (light green) channels. Individual data points and mean ± SEM are shown

We next examined the ability of MgADP to stimulate channel activity. Figure 2a,b shows that 100 μmol/l MgADP increased both wild-type and mutant K_ATP_ current amplitudes when added in the presence of inhibitory ATP. The mean increases in the hetS118L and homS118L K_ATP_ currents were not significantly different from that of the wild-type current (Fig. 2c).

### Metabolic inhibition of hetS118L and homS118L channels was unaffected

**Fig. 3:**
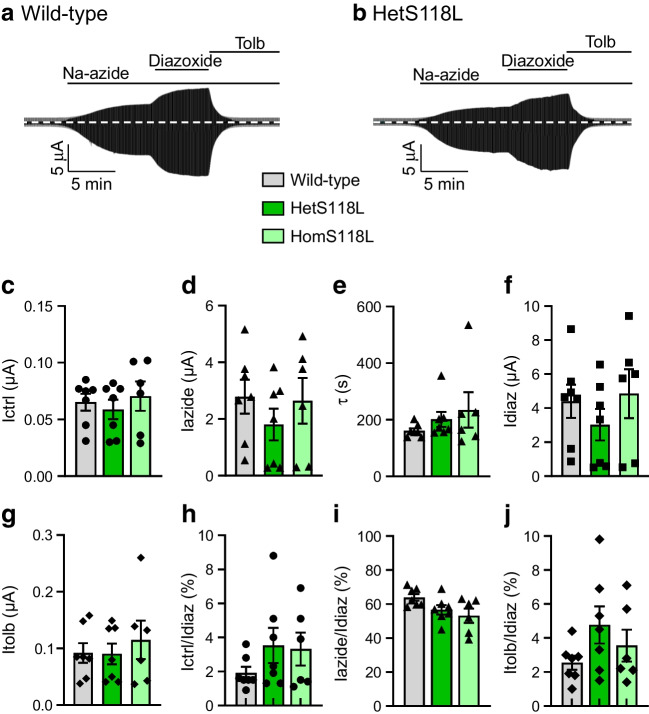
Effects of metabolic inhibition and diazoxide on K_ATP_ currents. (**a**, **b**) Representative whole-cell currents recorded from *Xenopus* oocytes expressing wild-type (**a**) or hetS118L (**b**) K_ATP_ channels in response to ±20 mV steps from a holding potential of −10 mV every ~2 s. The horizontal bars indicate when 3 mmol/l Na-azide, 340 µmol/l diazoxide or 0.5 mmol/l tolbutamide (Tolb) were added to the external solution. (**c**–**g**) Mean current amplitudes for wild-type (grey bars), hetS118L (dark green bars) and homS118L (light green bars) recorded in control solution (Ictrl, black circles) (**c**), and in the presence of Na-azide (Iazide, black triangles) (**d**), Na-azide+diazoxide (Idiaz, black squares) (**f**) or Na-azide+tolbutamide (Itolb, black diamonds) (**g**). (**e**) Time constants of K_ATP_ current activation by Na-azide. (**h**–**j**) Current amplitudes in control solution (**h**), and in the presence of Na-azide (**i**) or Na-azide+tolbutamide (**j**), expressed as a fraction of the diazoxide-activated K_ATP_ current. This controls for variability in K_ATP_ channel expression, assuming diazoxide causes maximal K_ATP_ channel opening in all cases. Box plots show individual data points and mean ± SEM (*n*=6 or 7). There was no significant difference between the data

To investigate the effects of the S118L mutation on the metabolic regulation of the K_ATP_ channel, we recorded whole-cell currents from *Xenopus* oocytes. As previously reported [24, 25], wild-type K_ATP_ channels were closed in control solution, due to the high intracellular ATP/ADP concentration, but were opened by lowering ATP/ADP using the metabolic inhibitor sodium azide (Fig. 3a,b). The current was further increased by the potassium-channel opener diazoxide (340 µmol/l) and almost fully inhibited by the sulfonylurea tolbutamide (500 µmol/l).

Mutations that reduce the channel ATP sensitivity normally increase the whole-cell current in both control solution and in the presence of azide, reflecting the fact that they are blocked to a lesser extent by the resting intracellular ATP concentration. Neither the hetS118L nor homS118L current amplitude was appreciably different from that of the wild-type current, either in control solution or in the presence of azide (Fig. 3a–d). This is consistent with the lack of a difference in ATP sensitivity (Fig. 1). The rate of current activation in response to azide was also similar (Fig. 3e). Diazoxide-activated wild-type, hetS118L and homS118L currents to similar extents (Fig. 3f), and tolbutamide blocked all three types of channel by ∼96% (Fig. 3g).

To control for variability in K_ATP_ channel expression between oocytes (and different batches of oocytes), we expressed currents in control solution (Fig. 3h), in azide solution (Fig. 3i) and in azide plus tolbutamide (Fig. 3j) as a percentage of that in the presence of diazoxide. Again, there was no difference between wild-type, hetS118L and homS118L currents.

### Membrane trafficking of S118L channels is impaired

**Fig. 4:**
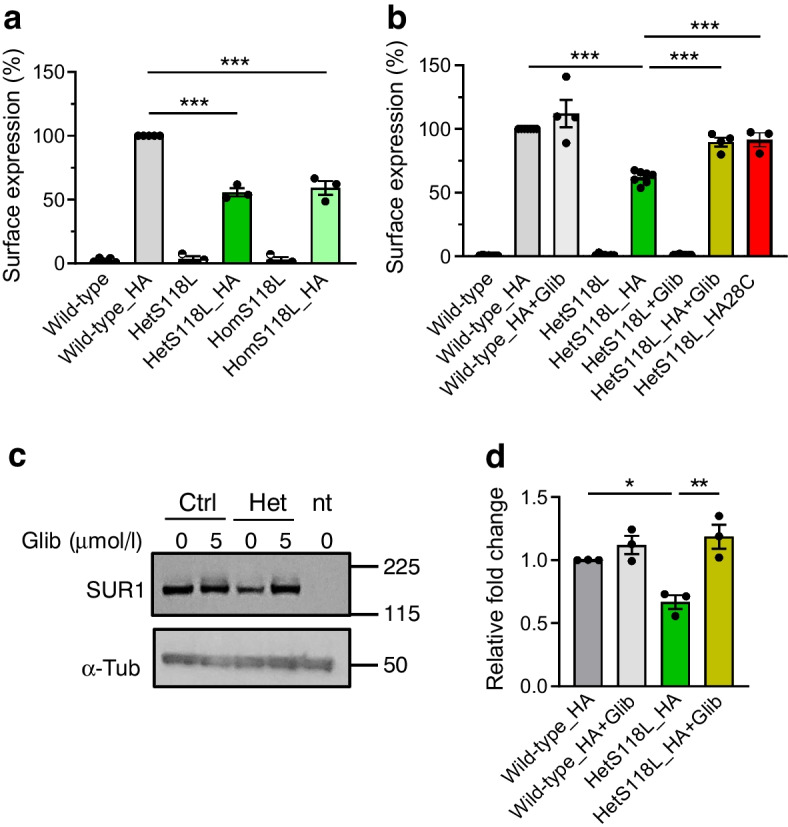
Glibenclamide rescues surface expression of hetKir6.2-S118L K_ATP_ channels. (**a**) Surface expression of HA-tagged or untagged SUR1 co-transfected with Kir6.2 (wild-type, grey bar, *n*=5), hetKir6.2-S118L (hetS118L; dark green bar, *n*=3) or homKir6.2-S118L (homS118L; light green bar, *n*=3). (**b**) Surface expression of HA-tagged or untagged SUR1 co-transfected with Kir6.2 (wild-type, dark grey bar, *n*=7), or hetKir6.2-S118L (dark green bar, *n*=7) cultured at 37°C in the absence of drug. Glibenclamide (5 µmol/l) was added to the media to promote surface membrane trafficking of wild-type and mutant constructs (wild-type, HA+Glib, light grey, *n*=4; hetKir6.2-S118L,_HA+Glib, olive bar, *n*=4). HetKir6.2-S118L was cultured at 28°C in the absence of drug (red bar, *n*=3). (**c**) Representative western blot for total SUR1 (~180 kDa) and α-tubulin (~50 kDa) proteins, in total protein lysates from HEK cells expressing wild-type Kir6.2 (ctrl), hetKir6.2-S118L (het) co-expressed with SUR1-HA, or not transfected (nt). Molecular mass markers shown on the right side of the blots are in kDa. (**d**) Quantification of the SUR1 bands, shown as a relative fold change against wild-type. Box plots show individual data points and mean ± SEM from three to seven independent transfections. **p<0.05, **p<0.01, ***p<0.001 (one-way ANOVA followed by the post hoc Dunnett’s test for multiple comparisons)

Because the ATP sensitivity and metabolic regulation of the mutant channels were unaltered, we explored if the mutation decreased the channel density in the plasma membrane. We used an HA tag, inserted into an extracellular epitope of SUR1, to detect K_ATP_ channels at the plasma membrane of transfected HEK293T cells. Surface expression was normalised first to protein content and then plotted as a percentage of the surface expression of the wild-type channel. No surface expression was detected in the absence of the HA tag. (Fig. 4a) There was an ~40% reduction in the surface expression of hetS118L and homS118L HA-tagged channels compared with wild-type channels (Fig. 4a), suggesting that the mutation disrupts surface trafficking and/or assembly of the K_ATP_ channel complex. However, membrane trafficking of hetS118L channels was partially rescued when cells were cultured in the presence of the sulfonylurea glibenclamide (5 µmol/l; olive bar, *n*=4, Fig. 4b) or at a lower temperature (28°C; red bar, *n*=3, Fig. 4b).

We also performed western blotting for SUR1 in total protein lysates from HEK cells expressing wild-type Kir6.2 or hetKir6.2-S118L co-expressed with SUR1-HA (Fig. 4c,d). Total SUR1 protein in hetKir6.2-S118L/SUR1-HA cells was less than in wild-type cells, consistent with the decrease in surface expression of the mutant channel. Glibenclamide co-culture corrected this effect of the mutation and increased expression of mutant channels.

## Discussion

We describe a female patient who presented with glucose intolerance at the age of 31 years and who subsequently progressed to gestational and early-onset insulin-independent, anti-islet-antibody-negative non-obese diabetes. Her presentation suggested a diagnosis of MODY, and genetic screening identified a dominantly inherited mutation in *KCNJ11*, S118L. Surprisingly, functional analysis of this mutation indicated that it is not a gain-of-function mutation but a loss-of-function mutation that impairs surface expression of the K_ATP_ channel by ~40%. Thus, our data provide support for the idea [14–16, 20–23] that dominantly inherited loss-of-function K_ATP_ channel mutations may lead to glucose intolerance and diabetes.

Loss-of-function mutations that cause a similar reduction in K_ATP_ channel density are usually associated with congenital hyperinsulinism not diabetes. However, the penetrance of dominantly inherited loss-of-function K_ATP_ channel mutations is often weak and some carriers may have no symptoms or undiagnosed hyperinsulinism in childhood [14]. Furthermore, many show spontaneous remission of symptoms [14–23]. This may explain why our patient was not diagnosed with hyperinsulinism.

Several possibilities might account for our patient’s diabetes. First, her diabetes may be caused by an unidentified (novel) MODY gene. Second, she may carry a combination of gene variants that predispose to non-obese type 2 diabetes and the additional stress of pregnancy precipitated her diabetes. Both explanations assume her *KCNJ11* mutation is an independent phenomenon without functional or clinical significance. Given the marked reduction in K_ATP_ channel density this seems unlikely. A third possibility is that her Kir6.2-S118L mutation did not cause symptomatic hyperinsulinism/hypoglycaemia in childhood but predisposed to glucose intolerance and diabetes in early adult life. We consider this explanation to best account for all the data. There is accumulating evidence in favour of the idea.

A progressive reduction in hypoglycaemia in patients with recessive inactivating K_ATP_ channel mutations is relatively frequent [14, 17–19] and in some individuals may evolve to glucose intolerance or, more rarely, diabetes [15, 16, 20–23]. The clinical history of our patient resembles that of individuals with the *ABCC8*-E1506K mutation, several of whom had gestational diabetes that remitted after pregnancy but subsequently returned [15, 16]. All *ABCC8*-E1506K carriers had low insulin values during a hyperglycaemic glucose clamp, indicating they had impaired insulin secretion. Likewise, individuals with another partial loss-of-function *ABCC8* mutation (R1353H) have been diagnosed with hyperinsulinism [28, 29], gestational diabetes [29, 30] and non-obese antibody-negative early-onset diabetes [30]. However, whether the progression of hyperinsulinism to glucose intolerance is a general feature of the disease is a controversial area [13] and one that warrants further investigation.

Interestingly, the phenotype is shared with certain *HNF4A* (MODY1) and *HNF1A* (MODY4) mutations that cause hyperinsulinaemia at birth before evolving to decreased insulin secretion and diabetes in later life [31–33]. It has been proposed that the hyperinsulinaemia is due to downregulation of K_ATP_ channel genes [33], suggesting a common mechanism may be involved. Further evidence is needed to substantiate this hypothesis. However, if it is correct, it becomes somewhat semantic as to whether patients like ours, who have reduced K_ATP_ channel expression associated with diabetes in early adult life, should be classified as having a novel type of MODY (caused by her loss-of-function K_ATP_ channel mutation), or congenital hyperinsulinism.

Why a partial reduction in K_ATP_ conductance should lead to glucose intolerance or diabetes is still not understood. However, it is consistent with mouse models of hyperinsulinism. Partial genetic deletion of either Kir6.2 or SUR1 causes increased excitability and insulin secretion [34, 35]. However, total knockout does not result in neonatal hypoglycaemia but instead dramatically reduces insulin secretion and causes impaired glucose tolerance in adult mice that progresses to overt diabetes when fed a high-fat diet [36–38]. A similar phenotype is seen when human loss-of-function mutations are expressed in mice [39, 40]. Various hypotheses have been put forward to account for these paradoxical findings but none has yet been substantiated [41].

### Mechanism of action of the S118L mutation

Both hetS118L and homS118L channels show normal ATP sensitivity and metabolic regulation, indicating that the mutation does not alter channel function. Instead, they reduce plasmalemmal K_ATP_ channel density. Multiple steps are involved in the surface expression of the K_ATP_ channel. Kir6.2 and SUR1 must be correctly translated, folded and co-assembled to form a hetero-octameric complex, which must be correctly trafficked to, and inserted into, the plasma membrane. A mutation may affect any of these processes, as well as the rate at which the channel is removed from the membrane. Neither Kir6.2 nor SUR1 traffic to the plasmalemma in the absence of the partner subunit, as they fail to exit the endoplasmic reticulum [27] and are then degraded [12]. The reduction in total SUR1 protein we observed favours the idea that channel assembly is affected, preventing surface trafficking. That surface expression was reduced to the same extent for hetS118L and homS118L channels is not unexpected. Given the tetrameric nature of the pore, the location of S118 at the interface between two pore subunits, and the known cooperativity between pore subunits, assembly of the whole tetramer is likely to be impaired by mutation of a single Kir6.2 subunit. Without correct assembly, SUR1 will be removed by the degradative machinery.

**Fig. 5:**
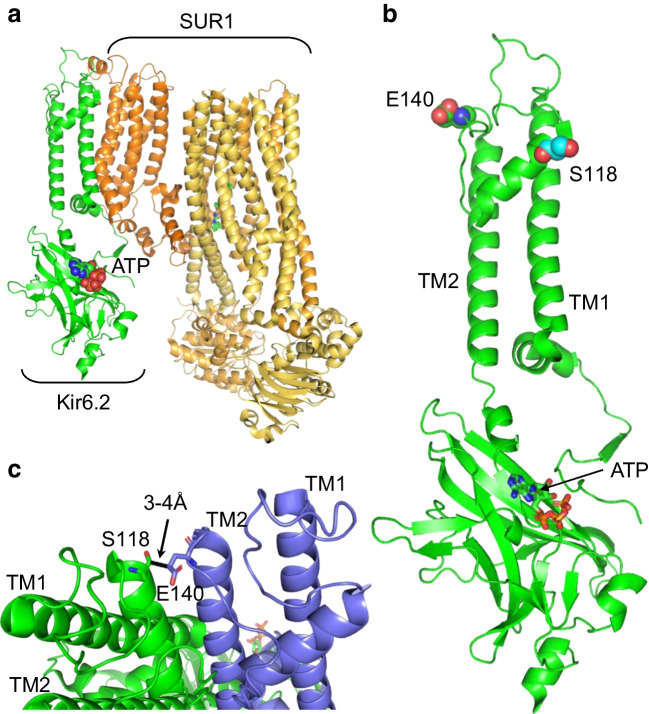
Location of S118L in Kir6.2. (**a**) Structural model of one pore-forming (Kir6.2, green) and one regulatory (SUR1, orange/yellow) subunit of K_ATP_ in an inhibited state (from Protein Data Bank [PDB] accession no. 6baa), with ATP bound to Kir6.2 and glibenclamide (not visible) bound to SUR1. (**b**) Side view, showing the position of S118 (cyan) at the start of the pore helix and E140 (green) at the top of transmembrane domain 2 (TM2). (**c**) Detail of two adjacent Kir6.2 subunits (one green, one blue) showing the close proximity (<4 Å) between S118 from one subunit with E140 from the neighbouring subunit

Structural studies of K_ATP_ channel open and closed states [42–45] reveal that S118 sits at the start of the pore helix, 3–4 Å from the adjacent Kir6.2 subunit (Fig. 5). However, it lies a significant distance from SUR1 (>14 Å), the closest approach being with the first set of transmembrane domains (TMD0) of SUR1. Thus, it is possible that the mutation affects channel assembly by disrupting interactions between Kir6.2 subunits and, thereby, indirectly influences interactions between Kir6.2 and SUR1. Interestingly, a heterozygous in-frame deletion of two adjacent residues, S116 and F117, led to a phenotype similar to that we report [23].

### Glibenclamide correction of surface expression

Our data indicate that glibenclamide acts as a pharmacological chaperone, enhancing surface expression of mutant channels. Likewise, reduced temperature enhanced surface expression. Both have previously been reported for hyperinsulinism-inducing mutations in *ABCC8* [10–12, 43]. Structural studies show the TMD0 of SUR1 (SUR1-TMD0) and the first transmembrane domain (TM1) of Kir6.2 make direct physical contact, explaining why SUR1-TMD0 mutations disrupt assembly and trafficking [46]. It has been proposed that this interaction is stabilised by pharmacological chaperones like glibenclamide, thus providing more time for SUR1-TMD0 and Kir6.2 to interact during channel assembly [47]. This may be how glibenclamide corrects assembly and trafficking of Kir6.2-S118L mutant channels.

### Conclusions

We report a novel loss-of-function mutation in *KCNJ11* (encoding Kir6.2) that results in a clinical phenotype largely indistinguishable from common gestational and type 2 diabetes, and was only identified by genetic screening. Because of the nature of the mutation our patient carried it was possible to manage her diabetes with glibenclamide and oral semaglutide rather than insulin injection, a significant advantage to the patient. Similarly, the diabetes of another patient, with a heterozygous loss-of-function mutation in SUR1, who progressed from hyperinsulinism to diabetes, was treatable with glibenclamide [16].

Determination of the cause of atypical diabetes is essential for proper management and understanding of possible syndromic effects. Cascade testing of family members, when there is a family history of diabetes, is essential and children at risk should be offered testing or monitoring following discussions with the family. Understanding the impact of variants of uncertain significance may require both functional and clinical studies, in addition to genetic studies. Individuals who present with diabetes in early adult life might have had undetected hyperinsulinaemia or diabetes in the neonatal period, leading to a misdiagnosis and potentially the wrong choice of therapy.

## Data Availability

The datasets generated during the current study are available from the corresponding author.

## Abbreviations

HA: Haemagglutinin
het: Heterozygous
HI: Hyperinsulinism
hom: Homozygous
IC_50_: Concentration producing half-maximal block of the K_ATP_ current
K_ATP_ channel: ATP-sensitive potassium channel
Kir6.2: Inwardly rectifying potassium channel 6.2
SUR1: Sulfonylurea receptor 1
TMD0: First set of transmembrane domains of SUR1
